# Predicting cardiovascular disease risk using retinal optical coherence tomography imaging

**DOI:** 10.3389/frai.2025.1624550

**Published:** 2025-11-18

**Authors:** Cynthia Maldonado-Garcia, Rodrigo Bonazzola, Enzo Ferrante, Thomas H. Julian, Panagiotis I. Sergouniotis, Nishant Ravikumar, Alejandro F. Frangi

**Affiliations:** 1 Centre for Computational Imaging and Simulation Technologies in Biomedicine, School of Computing, University of Leeds, Leeds, United Kingdom; 2 Research Institute for Signals, Systems and Computational Intelligence, sinc(i), Consejo Nacional de Investigaciones Científicas y Técnicas – Universidad Nacional del Litoral (CONICET-UNL), Santa Fe, Argentina; 3 Division of Evolution, Infection and Genomics, Faculty of Biology, Medicine, and Health, School of Biological Sciences, University of Manchester, Manchester, United Kingdom; 4 Manchester Royal Eye Hospital, Manchester University NHS Foundation Trust, Manchester, United Kingdom; 5 Manchester Centre for Genomic Medicine, Saint Mary's Hospital, Manchester University NHS Foundation Trust, Manchester, United Kingdom; 6 European Molecular Biology Laboratory, European Bioinformatics Institute (EMBL-EBI), Cambridge, United Kingdom; 7 Division of Informatics, Imaging, and Data Sciences, Faculty of Biology, Medicine, and Health, School of Health Sciences, University of Manchester, Manchester, United Kingdom; 8 Faculty of Science and Engineering, School of Computer Science, University of Manchester, Manchester, United Kingdom; 9 Christabel Pankhurst Institute, University of Manchester, Manchester, United Kingdom; 10 NIHR Manchester Biomedical Research Centre, Manchester Academic Health Science Centre, Manchester, United Kingdom

**Keywords:** optical coherence tomography, cardiovascular diseases, multimodal, deep learning, variational autoencoder

## Abstract

**Introduction:**

Cardiovascular Diseases (CVD) are the leading cause of death globally. Non-invasive, cost-effective imaging techniques play a crucial role in early detection and prevention of CVD. Optical Coherence Tomography (OCT) has gained recognition as a noninvasive method of detecting microvascular alterations that might enable earlier identification and targeting of at-risk patients. In this study, we investigated the potential of OCT as an additional imaging technique to predict future CVD events.

**Methods:**

We analyzed retinal OCT data from the UK Biobank. The dataset included 612 patients who suffered a Myocardial Infarction (MI) or stroke within five years of imaging and 2,234 controls without CVD (total: 2,846 participants). A self-supervised deep learning approach based on Variational Autoencoders (VAE) was used to extract low-dimensional latent representations from high-dimensional 3D OCT images, capturing structural and morphological features of retinal and choroidal layers. These latent features, along with clinical data, were used to train a Random Forest (RF) classifier to differentiate between patients at risk of future CVD events (MI or stroke) and healthy controls.

**Results:**

Our model achieved an AUC of 0.75, sensitivity of 0.70, specificity of 0.70, and accuracy of 0.70. The choroidal layer in OCT images was identified as a key predictor of future CVD events, revealed through a novel model explainability approach.

**Discussion:**

Our findings demonstrate the potential of retinal OCT imaging, when combined with advanced deep learning methods, as a predictive tool for identifying individuals at increased risk of CVD events.

## 1 Introduction

Cardiovascular Diseases (CVDs) continue to pose a significant global health challenge, affecting more than 500 million individuals worldwide. Specifically, in 2021, CVDs led to 20.5 million deaths. It is concerning to observe that up to 80% of premature Myocardial Infarction (MI) and stroke cases could potentially be prevented if detected early. Moreover, the burden of CVDs disproportionately impacts low- and middle-income countries, where nearly four out of every five CVDs-related deaths worldwide occur (Lindstrom et al., 2023). Currently, tools like the third version of the QRISK cardiovascular disease risk prediction algorithm (QRISK3) are utilized in primary care settings by healthcare professionals to pinpoint patients at higher risk of various CVDs. These tools are commonly employed during health assessments to assess a patient's risk based on factors such as demographic details (e.g., ethnicity, age, sex), clinical indicators (e.g., cardiac volume measurements, blood markers, indicators of obesity, etc.), and socioeconomic data (Li et al., 2019), but they require clinical infrastructure and laboratory testing, which may be limited in resource-constrained settings. This underscores the need for scalable, low-cost screening methods. Retinal optical coherence tomography (OCT) offers a promising solution: it is non-invasive, relatively inexpensive, and increasingly available in optometry and primary care settings. Retinal and choroidal microvasculature are sensitive indicators of systemic vascular health and have been linked to cerebral and coronary vascular disease. Thus, retinal imaging provides a unique window into microvascular health and enables the early identification of individuals at risk of CVD (Wong et al., 2022; Farrah et al., 2020; Chew et al., 2025; Arefin, 2025). Leveraging retinal imaging as a point-of-care screening tool could support overburdened health systems by enabling opportunistic risk assessment during routine eye exams and triaging high-risk patients for further cardiovascular evaluation.

Numerous prior studies have investigated the application of Artificial Intelligence (AI) in predicting CVDs risk factors using fundus photographs (Poplin et al., 2018; Nusinovici et al., 2022), as well as CVDs events (Diaz-Pinto et al., 2022; Cheung et al., 2020; Abdollahi et al., 2024). While retinal fundus photographs provide a two-dimensional surface view of the retinal vasculature, they are limited in their ability to capture information about the deeper retinal and choroidal layers. In contrast, OCT offers high-resolution three-dimensional cross-sectional imaging, enabling precise quantification of retinal layer thicknesses, choroidal architecture, and microstructural integrity. This depth-resolved information allows the detection of subtle microvascular changes that may precede clinically visible alterations on fundus photography, facilitating earlier detection of disease indicators and more precise monitoring of progression (Farrah et al., 2020). Furthermore, the volumetric nature of OCT data supports robust extraction of quantitative biomarkers, providing richer input for machine learning models and potentially improving risk stratification performance. This technological advancement in OCT has revolutionized retinal imaging by facilitating visualization of the chorioretinal microcirculation, which can serve as an early sign of microvascular disease. However, there remains limited research utilizing OCT as a predictor of CVDs (Maldonado-Garcia et al., 2022; Zhou et al., 2023).

This study introduces a predictive model that integrates features derived from 3D OCT imaging through a self-supervised deep neural network, along with patient demographic and clinical details. The aim is to detect individuals at risk of MI or stroke within five years after image capture. To the best of our knowledge, this is among the first investigations applying 3D OCT imaging and artificial intelligence for automatically forecasting patients vulnerable to adverse CVD incidents. The model advances the field in several ways employing a self-supervised Variational Autoencoder (VAE) combined with a multimodal Random Forest (RF) classifier to effectively integrate diverse patient data, and it incorporates an innovative vector-field-based explainability method to localize retinal features that contribute most to risk prediction, and it identifies the choroidal layer as a key predictive feature for cardiovascular risk.

## 2 Materials and methods

### 2.1 Database

**Figure 1:**
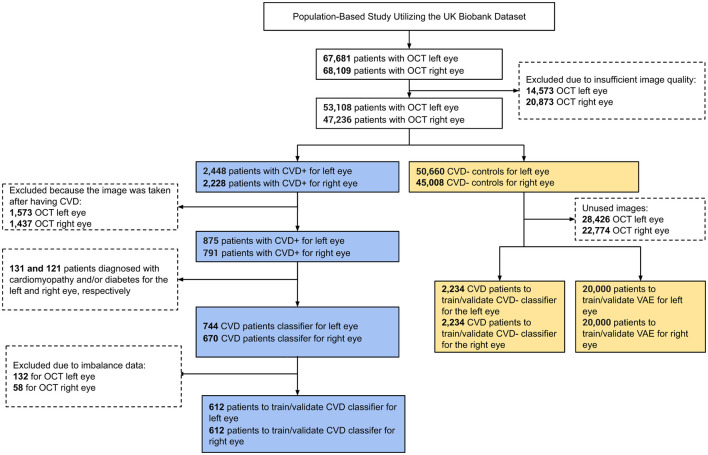
STROBE flow chart describing participant inclusion and exclusion criteria applied to define the study cohort.

In this research, we utilized retinal OCT imaging data sourced from the UK Biobank, captured using the Topcon 3D OCT 1000 Mark 2 system. OCT, a non-invasive imaging technique, employs light waves to generate intricate images of ocular structures like the retina, choroid, and optic nerve. The imaging procedure occurred in a dimly lit environment without pupil dilation, utilizing the 3D 6 × 6 *mm*^2^ macular volume scan mode, which consists of 128 horizontal B-scans arranged in a 6 × 6 mm raster pattern. The UK Biobank contains a vast array of health-related information from more than 500,000 participants in the UK, encompassing genetics, demographics, clinical measurements, lifestyle aspects, and medical imaging. Specifically, during their baseline visit [Instance 0, “Initial assessment visit (2006–2010)”], a total of 68,109 and 67,681 participants underwent retinal imaging for their right and left eyes, respectively. To ensure only high-quality images were included, we automatically evaluated image quality using a Quality Index (QI) detailed in a prior study (Stein et al., 2006). This QI is a globally accurate quality assessment algorithm derived from the intensity ratio, which is based on a histogram covering the entire image, and the tissue signal ratio, indicating the ratio of highly reflective pixels to less reflective ones. We excluded images representing the lowest 20% as indicated by the QI indicator, resulting in the exclusion of 14,573 images for the left eye and 20,873 images for the right eye, leaving 53,108 and 47,236 remaining images, respectively. Among these, we identified 2,448 (left eye) and 2,228 (right eye) images from participants who had experienced a stroke or MI event, referred to as CVD+ participants. However, only images from the left eye of 875 participants and the right eye of 791 participants were taken before the CVD event. Furthermore, we omitted 131 patients with diabetes and/or cardiomyopathy for the left eye and 121 patients for the right eye to minimize potential confounding, as both conditions are associated with retinal microvascular and choroidal alterations that could obscure associations specific to future myocardial infarction or stroke risk (Diaz-Pinto et al., 2022; Rakusiewicz et al., 2021; Yu et al., 2023). We also removed cases to address data imbalance, resulting in a final cohort of 612 subjects for both eyes. A visual representation of the participant selection and exclusion criteria used to establish the cohort for this study is depicted as a STROBE diagram in Figure 1. This study exclusively encompasses patients who experienced MI or stroke within a five-year period after OCT image acquisition. The acquisition details of reported CVD incidents' sources are elaborated in Supplementary Figure S1.

The size of the CVD+ group was determined based on the application of specific inclusion and exclusion criteria, as outlined in the STROBE diagram in Figure 1, resulting in a final count of 612 participants with OCT images of both eyes. For the non-CVD group or CVD-, 2,234 participants were propensity score-matched based on sex and age for OCT images of both eyes. The essential patient characteristics used to match the CVD+ and CVD- groups included demographic factors and clinical measurements, which are detailed in Table 1. The average age of individuals with and without CVDs was 60.78 ± 6.47 years, showing no significant difference between the two groups. The majority of participants in the UK Biobank cohort were of white ethnicity, with similar proportions in both groups. The average Body Mass Index (BMI) was 28.31 ± 4.45 kg/m^2^ for those with CVDs and 27.43 ± 4.33 kg/m^2^ for those without. In terms of blood pressure readings, individuals with CVDs had a Systolic Blood Pressure (SBP) of 147.26 ± 19.57 mm Hg, while those without CVDs had an SBP of 145.1 ± 18.75 mm Hg. The Diastolic Blood Pressure (DBP) was 84.75 ± 10.23 mm Hg for individuals with CVDs and 83.22 ± 9.73 mm Hg for those without. The mean level of Hemoglobin A1c (HbA1c) was 36.52 ± 4.32 mmol/L for individuals with CVDs and 36.59 ± 6.61 for those without. A notable percentage of participants reported being current alcohol consumers, accounting for 90.69% of individuals with CVDs and 91.83% of those without. These participant characteristics for both groups are summarized in Table 1.

**Table 1:** Characteristics of patients in the CVD+ and CVD- sets.

**Characteristics**	**CVD+**	**CVD-**
Number of subjects	612	2,234
Age: Mean (s.d), years	60.78 (6.47)	60.78 (6.47)
Sex: F, M %	29.74, 70.26	29.74, 70.26
Ethnicity, %	90.18 White, 4.26 Mixed, 3.93 Asian or Asian British, 0.33 Black or Black British, 0.16 Chinese, 1.15 Other ethnic group	89.22 White, 4.25 Mixed, 4.41 Asian or Asian British, 0.82 Black or Black British, 0.49 Chinese, 0.82 Other ethnic group
BMI: Mean, kg/m^2^	28.31 (4.45)	27.43 (4.33)
SBP: Mean, mm Hg	147.26 (19.57)	145.1 (18.75)
DBP: Mean, mm Hg	84.75 (10.23)	83.22 (9.73)
HbA1c: Mean, mmol/mol	36.52 (4.32)	36.59 (6.61)
HbA1c: %	2.43	2.44
Alcohol consumption: N, P, C, NA %	3.59, 5.72, 90.69, 0	3.92, 3.92, 91.83, 0.33

N, Never; P, Previous; C, Current; NA, Not answer.

**Figure 2:**
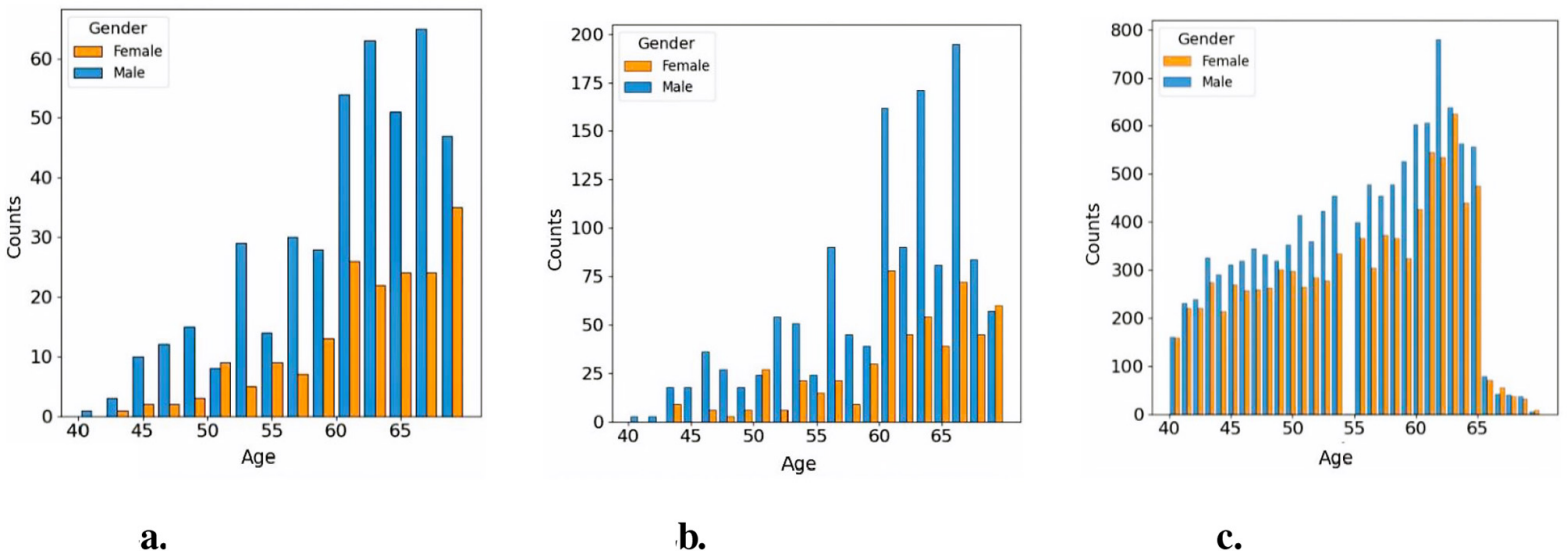
Age distribution across study cohorts. **(a)** Histogram of CVD+ participants included in the classification cohort, **(b)** Histogram of CVD- participants included in the classification cohort, **(c)** Histogram of CVD- participants included in the pretraining cohort.

We used an age-sex-matched cohort for the two groups. The relative proportion of patients included in the study in the two groups were 1:3 in the CVD and non-CVD group respectively. We used a 1:3 case:control matching ratio to maximize statistical power while keeping computational requirements tractable. Utilizing an age-sex-matched study cohort offers a significant advantage by helping to mitigate the influence of confounding variables that could bias the predictive model (Zhou et al., 2023). For instance, since age and sex are known to impact the risk of developing CVDs, matching groups based on these characteristics ensures that any observed differences in CVDs incidence are more directly associated with the retinal features, which are the variables of interest in this study. Previous research has indicated that matching on age and sex in the UK Biobank reduces variability within groups and enhances homogeneity among participants, thereby potentially improving the statistical power of the study (Batty et al., 2019, 2020). Furthermore, machine learning models may capture spurious correlations (i.e. to learn shortcuts) between predictors and targets, such as, for example, linking age or sex to the presence of pathology, unless care is taken when defining the predictors and study cohort (Brown et al., 2022). Figure 2 illustrates the age and sex in the CVD+ and CVD- patient groups, which were used to train and evaluate the predictive model proposed in this study. The construction of the metadata incorporated eight clinical variables, namely sex, age, HbA1c, systolic and diastolic blood pressure, alcohol consumption, and body mass index. The decision was made to omit the smoking variable from the study analysis because a significant number of participants did not provide responses to the relevant questionnaire item. From now on, the term “metadata” will be used to describe patient details, including demographic and clinical history information.

### 2.2 Framework of self-supervised feature selection variational autoencoder and multimodal random forest classification

**Figure 3:**
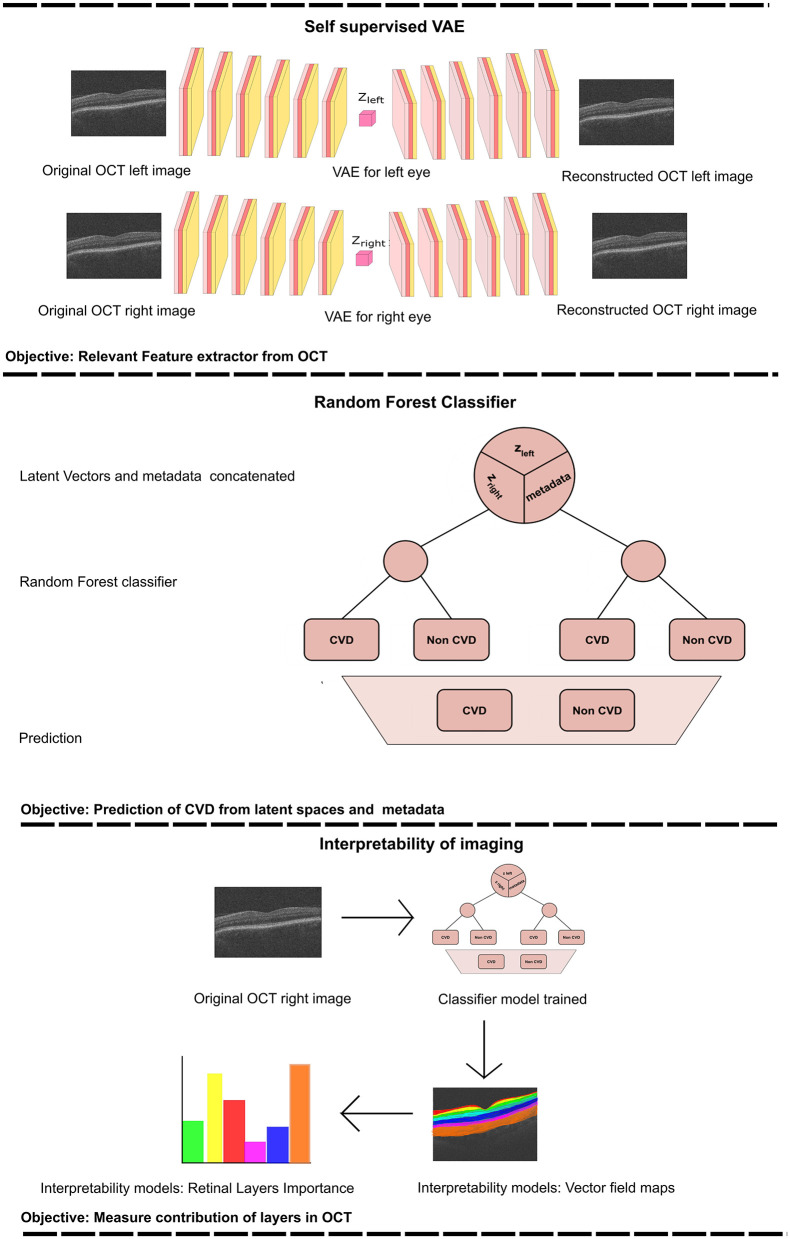
The provided workflow diagram illustrates the comprehensive process of training the Variational Autoencoder (VAE) and subsequently using it to acquire the latent vectors (upper section). These latent vectors are then combined with metadata and serve as inputs to the Random Forest (RF) classifier (middle section). Finally, we perform an interpretability analysis by perturbing the most relevant features, reconstructing the corresponding image and computing the vector field between the perturbed reconstructions (lower section). *z*_*left*_ represents the latent vector obtained from the training of the VAE for the left eye. *Z*_*right*_ corresponds to the latent vector acquired from training the VAE for the right eye.

This study proposes a predictive model for classifying patients into CVD+ and CVD- categories (Figure 3). The framework integrates a VAE (Kingma and Welling, 2013) for self-supervised feature extraction from retinal OCT images, and a RF classifier that combines the learned representations with patient metadata. The proposed model consists of two stages, a self-supervised feature extraction stage, and a subsequent classification stage.

#### 2.2.1 Self-supervised VAE

In the first stage of the proposed model, a VAE is used to learn latent representations for B-scan OCT images. VAE are a widely used type of generative model in neural networks, comprising a pair of networks or network branches that are trained together, called the encoder and decoder networks/branches. Given some input data (**x**), the encoder is designed to approximate the posterior distribution of the latent variables (*q*_ϕ_(**z**∣**x**)), under some assumed prior distribution (*p*(**z**)) over the latent variables (typically, a multivariate Gaussian prior is used, that is, p(z)~N(⋅)), while the decoder is trained to reconstruct the input data by sampling from the approximated posterior distribution $\left(p_{\theta }(\hat{x}|z\right)$. In other words, the encoder network maps inputs to low-dimensional latent representations, and the decoder network acts as the generative model. The approximation of the true but intractable posterior distribution is obtained by maximizing the Evidence Lower Bound (ELBO), which can be expressed as follows:


$$\begin{array}{l} \text{ELBO}=E_{q_{\phi }\left(\text{z}|\text{x}\right)}\left[\log p_{\theta }\left(\text{x}|\text{z}\right)\right]-D_{KL}\left(q_{\phi }\left(\text{z}|\text{x}\right)||p\left(\text{z}\right)\right) \end{array}$$ (1)

The loss function utilized for training the proposed self-supervised VAE comprises two key elements: (1) the loss of Mean Square Error (MSE) $L_{MSE}$, detailed in Equation 3, which evaluates the discrepancy in reconstruction between the original data (**x**_*i*_) and the reconstructed data (${\hat{\text{x}}}_{i}$), and (2) the loss of Kullback-Leibler divergence $L_{KL}$, illustrated in Equation 4. KL divergence quantifies the dissimilarity between the learnt latent distribution *q*(**z**) and a previously specified distribution *p*(**z**), which, in this scenario, the prior *p*(**z**) is a multivariate Gaussian distribution. The parameters of the learned distribution *q*(**z**) are its mean (μ_*i*_) and variance ($\sigma_{i}^{2}$). By minimizing the KL divergence, the model is incentivised to shape a latent space that adheres to the target Gaussian distribution. The integration of these two loss components steers the VAE toward the dual objective of reducing reconstruction errors and aligning the learnt latent distribution with the intended prior distribution.


$$\begin{array}{l} L_{VAE}=L_{MSE}+\beta L_{KL} \end{array}$$ (2)

$$\begin{array}{l} L_{MSE}=\frac{1}{N}\overset{N}{\underset{i=1}{\sum }}{\left({\text{x}}_{i}-\hat{{\text{x}}_{i}}\right)}^{2} \end{array}$$ (3)

$$\begin{array}{l} L_{KL}=\frac{1}{2}\left[1+\log\left(\sigma_{i}^{2}\right)-\sigma_{i}^{2}-\mu_{i}^{2}\right] \end{array}$$ (4)

Given the relatively small number of CVD events, training a fully supervised, well-known Convolutional Neural Network (CNN) could lead to overfitting. A self-supervised VAE mitigates this risk by learning compact latent representations from the full OCT dataset without outcome labels, capturing meaningful microstructural variability. By combining reconstruction and KL divergence losses, the VAE creates a structured latent space that captures informative patterns in retinal layers relevant to cardiovascular risk. These latent features can then be used by downstream classifiers to improve prediction performance compared to using clinical variables alone. Prior studies in medical imaging show that VAEs or autoencoder-based pretraining improve classification performance when labeled data are limited (Ehrhardt and Wilms, 2022; Rais et al., 2024).

For this study, we trained independent VAEs for each eye, to learn unique latent features from the OCT images. Subsequently, a classifier used these learnt features to predict the probability of an individual's prospective CVD incidence.

#### 2.2.2 Classification

Using the features acquired from the VAE in the previous stage, we trained a Random Forest (RF) classifier to distinguish between individuals in the CVD+ and CVD- categories, as illustrated in Figure 3. The input for this process consists of the latent vector representation of each OCT image generated by the VAE for each eye, which is merged with a vector containing the relevant patient information. RF are a type of ensemble machine learning method that involves multiple decision trees, each of which is trained on a randomly selected subset of training data (Breiman, 2001). Using the power of numerous decision trees and incorporating random feature selection, this ensembling technique enhances the generalisability of the predictive model to new data by reducing model variance by averaging predictions from the trees in the ensemble. RF have been widely applied in medical settings for both classification and regression tasks, including in previous studies related to CVD diagnosis (Khozeimeh et al., 2022; Yang et al., 2020). One notable advantage of RF compared to other classification algorithms is their ability to easily handle multimodal data that include various data types (such as categorical, ordinal, and continuous). Decision trees within the ensemble operate independently, and their combined predictions are aggregated to produce the final RF prediction for a given input using majority voting for classification tasks. This structure also provides feature importance, which enhances the explainability of the model's decisions. Additionally, RF is computationally efficient when compared to more complex models, such as neural networks, as it does not require GPU resources for training.

### 2.3 Experiment details

All experiments were carried out on an NVIDIA Tesla M60 GPU using PyTorch (v1.10.2). A grid search strategy with five-fold cross-validation was employed to determine the optimal hyperparameters (Supplementary Table S1). The dataset was divided into training, validation, and test sets in a 6:2:2 ratio. The encoder and decoder networks each consisted of six 2D convolutional layers; the encoder used Rectified Linear Unit (ReLU) activations, while the decoder used LeakyReLU activations (see Supplementary Table S2). Model training was performed in two phases: an initial self-supervised learning phase to acquire latent representations independently for each eye, followed by a fine-tuning phase initialized with the learned weights. Specifically, the left and right eye VAEs were trained independently from scratch. When training a model using both eyes simultaneously, we initialized the networks with the previously trained left/right VAE weights to reduce computational cost and accelerate convergence.

The VAE was trained with a latent space size of 128 using KL annealing to improve training stability. The KL divergence weight (β) was gradually increased from 0.001 at the start of training to a maximum of 0.01 over the first 20 epochs, after which it was held constant for the remaining training epochs, allowing the model to prioritize reconstruction early on before encouraging disentangled latent representations (Ichikawa and Hukushima, 2024). The VAE was trained for 100 epochs in total. Other hyperparameters, including CNN channels, batch size, learning rate, and weight decay, were optimized via grid search, with the tested and optimal values reported in Supplementary Table S1. Hyperparameter details for downstream Random Forest models are summarized in Supplementary Table S3.

During the classification phase, we conducted a thorough investigation into the impact of latent representations derived from the OCT images of the left and right eyes, along with patient metadata, on the predictive task. This was accomplished by generating seven datasets from the same group of 2846 patients, each comprising different combinations of data sources: (i) latent representations from the left eye only (LE); (ii) latent representations from the right eye only (RE); (iii) latent representations from both eyes (BE); (iv) metadata only (MTDT); (v) left eye with metadata (LE-MTDT); (vi) right eye with metadata (RE-MTDT); and (vii) both eyes with metadata (BE-MTDT). Random Forest classifiers were trained separately on each of these seven datasets, as depicted in Figure 3 for the BE-MTDT dataset. Finally, the optimal hyperparameter values of RF classifiers were determined through a combination of grid search and empirical experimentation, to identify the best performing RF model for each specific dataset (see Supplementary Table S3). We divided the dataset into training, validation, and test sets, following a distribution ratio of ~5:2:3, respectively (resulting, 1423 patients in the training set, 459 patients in the validation set and 964 patients in the held-out unseen test set). Grid search was performed using five-fold cross-validation, while an independent, unseen test set remained fixed throughout all experiments to evaluate all trained classifiers fairly. During the optimal hyperparameter search, we employed Recursive Feature Elimination (RFE) as a feature selection technique to address overfitting and train the model with the most pertinent variables for classification. In the majority of cases, the model was trained with the top 10 most significant features, with the exception of the RE case, where the optimal conditions involved using only 5 variables.

To evaluate the effectiveness of our model, we compared predictive performance against the QRISK3 algorithm, the current gold standard used by healthcare professionals / cardiologists to assess the patient's risk of stroke or heart attack, in a 10-year period. The QRISK3 score was calculated within the specified test dataset, following the methodological guidelines outlined in Li et al. (2019). The evaluation of the QRISK3 score involved entering essential variables which are described in Supplementary Table S4. In cases where certain information was missing, we consistently represented these gaps as “0” when evaluating the QRISK3 scores on the test dataset. For our classification task, we evaluated the model performance using a range of metrics. Accuracy, sensitivity, and specificity were determined by calculating true positives, true negatives, false positives, and false negatives (using a classification probability threshold of *t* = 0.5, i.e. if the predicted probability is ≥0.5, the patient is classified as CVD+, else as CVD-). The Area Under the Receiver Operating curve (AUROC) was then employed as a performance measure to assess both our model and the QRISK3 algorithm.

### 2.4 Model explainability

Predictive models based on machine/deep learning algorithms, proposed for identifying risk of disease from medical imaging often fail to report both “local” and “global” explanations for the model's predictions. This is especially prevalent in the case of deep learning-based approaches that are often treated as black boxes, with little information provided on the mechanism by which models arrive at specific predictions. Local explanations provide insights to individual decisions/predictions of the model. For example, this may involve identifying specific input variables/regions of an image that had the most influence on the model's prediction for that instance. On the other hand, global explanations describe the model's behavior across predictions for all instances in all classes of interest. Specifically, global explanations provide information on the most common discriminative features identified by the model for all instances in each class of interest. Providing both local and global explanations of model behavior is essential for developing responsible AI in healthcare applications, as it can help identify systematic biases in data and mitigate for the same (e.g., learning of “short-cuts” is a common issue encountered in the application of deep learning-based methods for predictive tasks using medical images), build trust in AI systems by improving transparency in model decision making, and may even provide new insights to previously known associations between image-derived phenotypes and the presence or progression of diseases. Therefore, in this study, we employ distinct techniques to provide both local and global explanations for the proposed predictive model.

To provide local explanations of the behavior of the model and elucidate how the model uses OCT imaging-derived features to classify instances in the CVD+ group, we first selected the best performing RF classifier according to AUC value. Subsequently, based on the selected RF classifier, we identified the latent variable derived from the OCT image with the highest importance assigned by the RF, which we denoted **z**_*max*_. To visually assess the regions of the retina in OCT images that contribute significantly to the prediction of CVD, we propose a novel vector field-based latent traversal approach that evaluates the impact of perturbing the most important latent feature **z**_*max*_ on subsequent reconstructions of OCT images. Specifically, given an image **x**, we compute the corresponding latent vector **z** using the trained encoder network. Next, we perturb only the dimension **z**_*max*_ of the calculated latent vector and reconstruct the corresponding image. This perturbation is performed by multiplying the latent dimension **z**_*max*_ by a scalar value, which in this case is the standard deviation of this latent component, calculated throughout the training population, defined as σ_*max*_. The remaining latent variables in the computed latent vector are left unchanged, resulting in a perturbed latent vector ($\hat{\text{z}}$). Finally, we reconstruct the input OCT image ($\hat{\text{x}}$) using the perturbed latent vector $\hat{\text{z}}$.

To visualize the regions in the reconstructed OCT images affected by the altered latent vector **ẑ**, we examine the differences between the initial image **x** and the reconstructed image $\hat{\text{x}}$ derived from $\hat{\text{z}}$. In this context, we calculate the vector field between these images using the Lucas-Kanade algorithm (Lucas and Kanade, 1981). The resulting vector field, showing the magnitude of the displacement vector for the moving pixels, was then superimposed on the original image to create a visual representation. The vector field indicates how the pixels between the images (that is, **x** and $\hat{\text{x}}$) change due to the latent traversal from **z** to $\hat{\text{z}}$. The estimated vector field between **x** and $\hat{\text{x}}$ helps to visually illustrate the regions in the OCT image that were altered by modifying the latent component **z**_*max*_. This aids in pinpointing the areas of the image influenced by alterations to the crucial latent variable for accurately classifying a patient's CVDs risk based on their OCT image(s), and consequently, helps to understand which retinal areas are informative for distinguishing between the CVD+ and CVD- patient groups.

To provide global explanations of the behavior of the model, we calculate the importance of the characteristics assigned to each characteristic by the RF in each predictive model investigated. As mentioned previously, the RF in each predictive model were trained using a reduced set of characteristics identified by RFE. Feature importance is calculated in RF as the average Gini information gain for any given feature, calculated across all decision trees in the forest. The feature importance values for all classifiers studied in this work, across all test set instances, are summarized as bar plots later. Additionally, we calculate the relative importance of the type / channel of data used as inputs/predictors in this study, namely, OCT images of the left and right eye and patient metadata, for the task of distinguishing between the CVD+ and CVD- groups.

## 3 Results

### 3.1 Classification performance

As discussed previously, we trained and evaluated the performance of several RF classifiers, where each classifier was trained and evaluated independently using seven different combinations of data types obtained from the same set of patients (comprising CVD+ and CVD- groups). Specifically, the datasets used were LE, RE, BE, LE-MTDT, RE-MTDT, BE-MTDT and MTDT. Henceforth, for brevity, we refer to classifiers trained and evaluated on these datasets as LE-RF, RE-RF, BE-RF, LE-MTDT-RF, RE-MTDT-RF, BE-MTDT-RF, and MTDT-RF. The performance of all seven classifiers was evaluated and compared using the same unseen test set (which contains 964 patient data, 834 CVD- and 130 CVD+), and using the same set of evaluation metrics outlined in Section 2.3. The rationale for comparing all seven classifiers against each other was to - (i) assess whether combining latent features learnt from OCT images of both eyes (BE) provided greater discriminative power than using those from either left (LE) or right eye (RE) alone; (ii) compare the discriminative power of OCT image-derived latent features against patient metadata; and (iii) evaluate the discriminative power gained by enriching OCT image-derived latent features with patient metadata.

The performance of all seven classifiers on the unseen test set is summarized in Table 2. These results show that the BE-MTDT-RF classifier consistently outperformed all six other classifiers, with statistically significant differences in *p*-values (refer to Supplementary Table S5). In terms of evaluating the effectiveness of OCT image-derived characteristics and metadata information for classifying CVD+ and CVD- patients, results for the BE-MTDT-RF, LE-MTDT-RF, RE-MTDT-RF and MTDT-RF classifiers indicate that combining latent features learnt from OCT images with patient metadata (i.e. BE-MTDT-RF, LE-MTDT-RF, RE-MTDT-RF), consistently improves classification performance relative to using patient metadata alone (MTDT-RF). Specifically, the BE-MTDT-RF classifier demonstrated the highest performance, achieving an accuracy of 0.70, sensitivity of 0.70, specificity of 0.70, and an AUC score of 0.75. Furthermore, combining latent features from RE or LE OCT images with metadata (i.e., LE-MTDT-RF and RE-MTDT-RF) resulted in improvements of 13 − 17% across all classification metrics relative to the MTDT-RF classifier. Notably, the MTDT-RF classifier exhibited the lowest values across all classification metrics, with an accuracy, sensitivity, and specificity of 0.52, and an AUC score of 0.57. The BE-RF, RE-RF, and LE-RF classifiers also consistently outperformed the MTDT-RF classifier in all classification metrics. However, they were outperformed by classifiers that incorporated OCT image-derived features with patient metadata (i.e., BE-MTDT-RF, LE-MTDT-RF, RE-MTDT-RF).

**Table 2:** Predictive analysis of Cardiovascular Disease (CVD) metrics utilizing UK Biobank data across seven distinct cases.

**Classification metrics**				
**Classifier**	**Accuracy (95% CI)**	**Sensitivity (95% CI)**	**Specificity (95% CI)**	**AUC (95% CI)**
LE-MTDT-RF	0.68 (0.65–0.71)	0.69 (0.66–0.73)	0.68 (0.65–0.72)	0.72 (0.69–0.75)
RE-MTDT-RF	0.67 (0.65–0.69)	0.69 (0.67–0.71)	0.67 (0.65–0.69)	0.70 (0.68–0.72)
**BE-MTDT-RF**	**0.70 (0.67–0.73)**	**0.70 (0.67–0.73)**	**0.70 (0.67–0.72)**	**0.75 (0.72–0.78)**
MTDT-RF	0.52 (0.50–0.54)	0.52 (0.50–0.54)	0.52 (0.50–0.54)	0.57 (0.55–0.59)
LE-RF	0.61 (0.58–0.64)	0.56 (0.53–0.59)	0.62 (0.59–0.65)	0.64 (0.61–0.67)
RE-RF	0.58 (0.56–0.60)	0.53 (0.51–0.55)	0.59 (0.57–0.61)	0.62 (0.60–0.64)
BE-RF	0.64 (0.62–0.67)	0.57 (0.55–0.59)	0.65 (0.63–0.68)	0.67 (0.64–0.70)

All metrics are reported as mean values with 95% confidence intervals (CI). Bold values indicate the highest performance for each classification metric.

**Figure 4:**
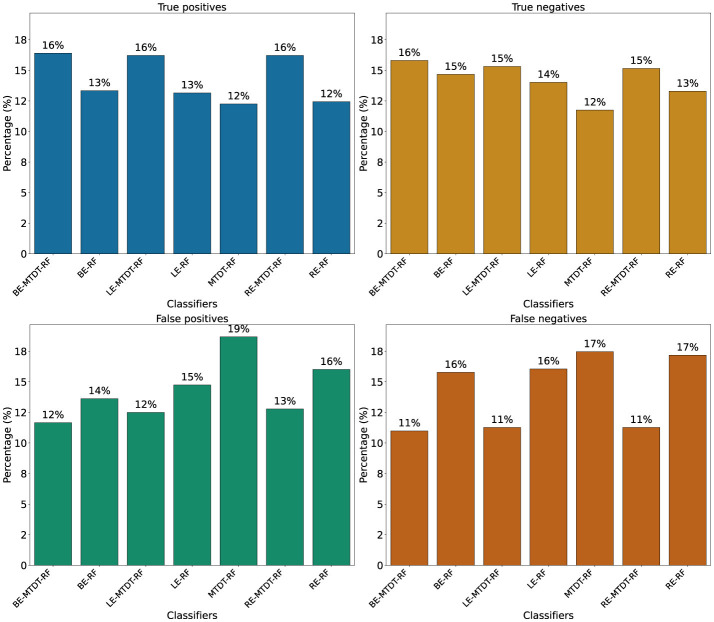
Comparison of classifier performance in terms of True Positives (TP), True Negatives (TN), False Positives (FP), and False Negatives (FN). Values above the bars represent the corresponding counts for each classifier. **(a)** True positives. **(b)** True negatives. **(c)** False positives. **(d)** False negatives.

Figure 4 presents four histograms depicting true positives (Figure 4a), true negatives (Figure 4b), false positives (Figure 4c), and false negatives (Figure 4d) for the seven classifiers investigated. A consistent observation across all our results is that the BE-MTDT-RF classifier misclassified fewer instances in the CVD+ group, than all other classifiers, which is consistent with the classification metrics summarized in Table 2. Similarly, the LE-MTDT-RF and RE-MTDT-RF classifiers exhibited good sensitivity by incurring few false negative errors percentages (11%) in the CVD+ group were incorrectly classified (Figure 4d). The MTDT-RF classifier yielded fewer true positives and true negatives (12% in both) compared to the other classifiers that utilized only OCT features (Figures 4a, b, respectively), which aligns with the results presented in Table 2.

**Figure 5:**
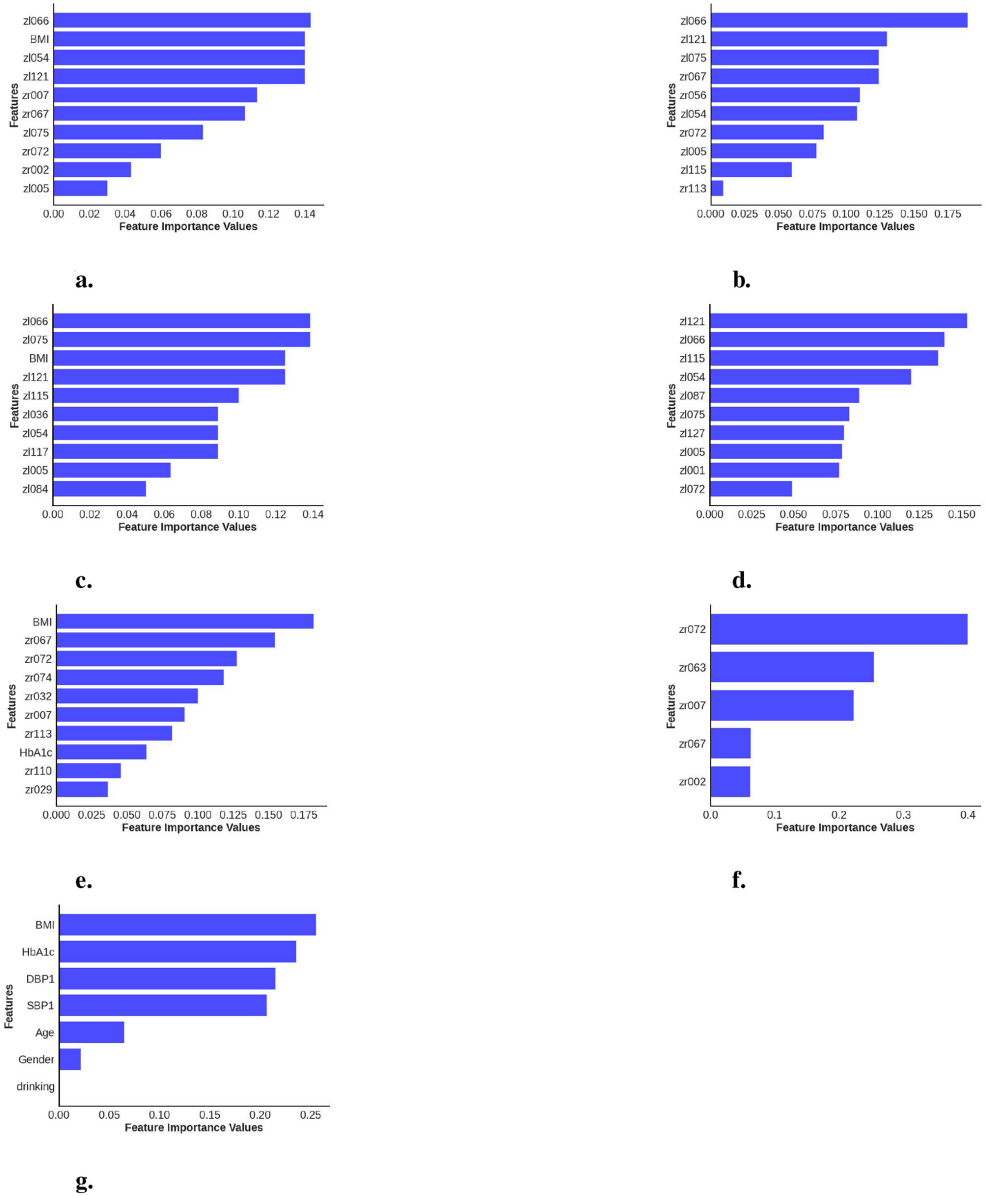
Calculation of feature importance magnitudes for the seven different classifiers investigated, where each classifier uses different combinations of data data channels/modalities. **(a)** BE-MTDT-RF. **(b)** BE-RF. **(c)** LE-MTDT-RF. **(d)** LE-RF. **(e)** RE-MTDT-RF. **(f)** RE-RF. **(g)** MTDT-RF.

A significant observation in the results is that including both eyes was advantageous for both cases, BE-MTDT-RF and BE-RF, compared to their counterparts, LE-MTDT-RF and RE-MTDT-RF, RE-RF, and LE-RF, respectively. Furthermore, in both scenarios, with/without metadata, the left eye consistently provided improved classification performance compared to the right eye. This finding was statistically significant, as indicated by the *p*-values reported in Supplementary Table S6. This observation is in concordance with the global explanations of models' predictions summarized in Figure 5, wherein features attributed to the left eye were found to be more discriminative (i.e., had higher feature importance) than those of the right eye. Confusion matrices and precision-recall curves for all classifiers are provided in Supplementary Figures S2, S3.

Subsequently, we conducted a comparative analysis of the best classifier identified from the previous experiments, namely BE-MTDT-RF, and the QRISK3 algorithm, the current clinical standard for assessing patients at risk of stroke or MI. In our age-sex matched UK Biobank cohort, BE-MTDT-RF achieved modestly higher discriminative performance (AUC: 0.75 vs. 0.60; Table 3).

**Table 3:** Comparison of classification metric results between our model employing both ocular data and metadata (BE-MTDT) and the QRISK algorithm.

**Classification metrics**				
**Modality**	**Accuracy (95% CI)**	**Sensitivity (95% CI)**	**Specificity (95% CI)**	**AUC (95% CI)**
**BE-MTDT-RF**	**0.70 (0.67–0.73)**	**0.70 (0.67–0.73)**	**0.70 (0.67–0.72)**	**0.75 (0.72–0.78)**
QRISK	0.55 (0.53–0.57)	0.60 (0.58–0.62)	0.55 (0.52–0.58)	0.60 (0.57–0.63)
χ^2^ = 95.72, *df* = 1, *p* = 1.31 × 10^−22^				

All metrics are reported as mean values with 95% confidence intervals (CI). McNemar's Test (*p < 0.005). Bold values indicate the highest performance for each classification metric.

### 3.2 Model explainability

In order to provide local explanations for the behavior of all classifiers investigated in this study, we analyzed the most important features identified by each model (refer to Figure 5) for distinguishing between the CVD+ and CVD- groups. Important features identified for the best performing classifier, namely, BE-MTDT-RF in particular, provided some noteworthy insights. As highlighted in Figure 5a, we found that a latent variable learned from the left-eye OCT image, denoted zl066, had the most influence on the classifier's ability to separate CVD+ and CVD- patient groups. Additionally, among the top 10 most important features identified for the BE-MTDT-RF classifier, 9 of the features pertained to latent variables learned from the left-eye OCT image. BMI was the only feature from the basic set of patient metadata used to train the classifier, that was identified to have a significant influence on the classifier's predictions. Furthermore, looking at the local explanations summarized in Figures 5b–d, we observe that latent variable zl066 consistently ranks among the top two most important features for the BE-RF, LE-MTDT-RF, and LE-RF classifiers, respectively. This indicates that the retinal features encoded by zl066 are consistently considered to be relevant across all four classifiers presented in Figures 5a–d. Among the classifiers which combined retinal OCT-image derived features with patient metadata, namely, BE-MTDT-RF, LE-MTDT-RF and RE-MTDT-RF, we observed that only two features, namely, BMI and HbA1c, ranked among the top 10 most important features for the classification task. Both features are known and established cardiovascular risk factors, and importantly, we infer from these results that the latent variables learned from the retinal OCT images, had a greater influence on the classifiers' predictions than the patient metadata variables.

**Figure 6:**
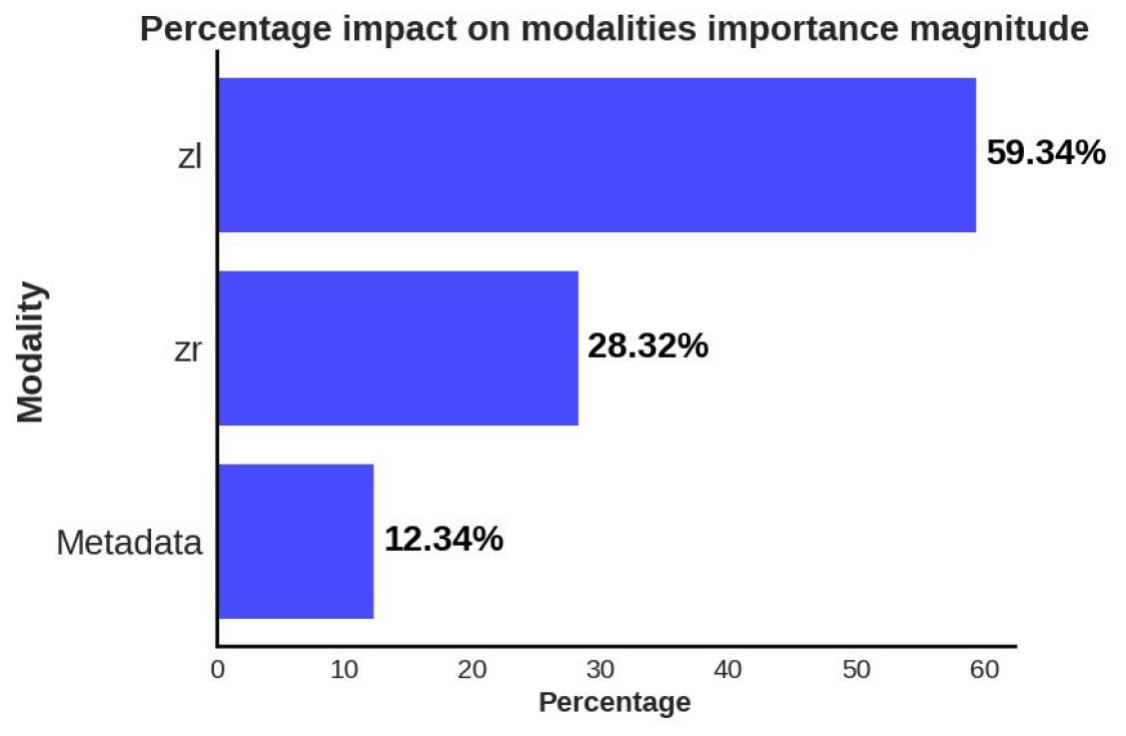
Global explanations of features from different data modalities/channels which were considered important by the predictive model for separating the CVD+ and CVD- groups. Bar plot summarizes the relative importance of latent variables from left (*zl*) and right (*zr*) eye OCT images and patient metadata, as percentages.

**Figure 7:**
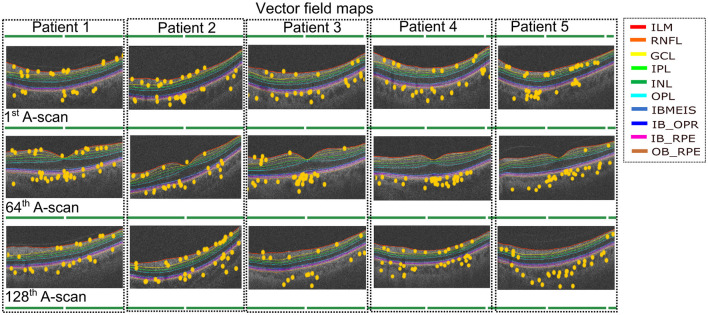
Vector field maps presented for three differents B-scans for the left eye. The top row corresponds to the 1st B-scan, the middle image for the 64th B-scan while the bottom row depicts the final B-scan. The yellow circles represent the regions that the vector field maps highlight when modifying the latent variable zl066. The legend includes the names of layer boundaries in the optical coherence tomography images, as follows: Internal Limiting Membrane; RNFL: Retinal Nerve Fiber Layer; GCL: Ganglion Cell Layer; IPL: Inner Plexiform Layer; INL: Inner Nuclear Layer; OPL: Outer Plexiform Layer; BMEIS: Boundary of Myoid and Ellipsoid of Inner Segments; IB_OPR: Inner Boundary of Outer Segment Retinal Pigment Epithelium Complex; IB_RPE: Inner Boundary of Retinal Pigment Epithelium; OB_RPE: Outer Boundary of Retinal Pigment Epithelium.

Using the insights gained from analyzing the global explanations of classifier behavior summarized in Figure 6, we propose a novel approach based on latent space traversals to translate the former into local explanations that provide insights to regions of the OCT image that contain relevant information for correctly identifying patients at risk of cardiovascular disease. Specifically, having identified latent variable zl066, derived from left-eye OCT images, as being the most important feature for classification, our local explainability approach (refer to Section 2.4) begins by perturbing the values of the latent variable for any image in the CVD+ group, reconstructs the OCT image using the perturbed latent representation (using the pretrained VAE) and then estimates vector field maps between the original and perturbed OCT image reconstructions, seeking to pinpoint the specific image regions that change as a result of the perturbation. We conducted a qualitative analysis by estimating the vector field maps between the original reconstructed B-scan OCT images and their perturbed counterparts for all CVD patients in the test set. By overlaying the estimated vector field map onto the original OCT images, we visualized the retinal image features encoded by the latent variable of interest. Although this process was applied to all high-risk CVD patients, we present results for five representative cases here (Figure 7), with each row representing one patient. We considered 3 B-scans per patient, including the 1st one (upper row), 64th (middle row) and 128th (bottom row). Finally, by overlaying the estimated vector field maps onto the original OCT images, we obtained visual interpretations of the retinal image features encoded by the latent variable of interest.

The vector field maps generated by our model highlighted the choroidal layer in the majority of B-scans, with additional identification of layers adjacent to the choroid, including the Retinal Pigment Epithelium (RPE). Additionally, the vector field maps highlighted the regions in the inner retinal layers, likely corresponding to the Retinal Nerve Fiber Layer (RNFL) and Ganglion Cell Layer (GCL) (Zhou et al., 2023). Although some other layers received some, if less, emphasis, the main focus remained on the choroidal layer and the innermost layers. The vector field maps provide precise localization of the image regions modeled by latent variable zl066 (visualized as landmarks, as shown in Figure 7), thereby providing local explanations for the most discriminative regions within the OCT, and providing insights to which retinal layers may contain relevant information for predicting risk of CVD in patients. In particular, these local explanations highlighted the relevance of information contained within the choroidal layer of the retina, for distinguishing between CVD+ and CVD- patient groups. To further validate the robustness of our findings, we applied an occlusion-based approach (Supplementary Figure S4), which produced qualitatively similar results. Notably, our vector field perturbation method highlights regions more consistently across the entire image and all B-scans. For instance, the choroid is captured as a whole rather than partially, as observed with the comparison method.

## 4 Discussion

Our findings indicate that the use of retinal OCT images in conjunction with VAE and multimodal RF classification has potential to identify patients at risk of CVDs (within a five-year interval). Our investigation included the deployment of a self-supervised VAE coupled with an RF classifier framework, which incorporates B-scan OCT images and metadata as distinct modalities. This integration allowed our model to discern the specific attributes within the OCT images that contribute significantly to the prediction of CVDs. Importantly, our study distinguishes itself by interpreting the particular OCT image features (at both the global i.e. class/category, and local i.e. instance, levels), which are relevant to the classification task and thereby provide insights to the key regions of the retinal image that are most discriminative. To the best of our knowledge, some studies have ventured into the application of OCT within a primary care framework for CVDs. However, these studies were limited in their explanatory capacity regarding the effects of including images from both eyes and different types of patient data, and used a small portion of the OCT B scans, limiting the information from the entire volume. Nevertheless, the performance results show promising outcomes for OCT as a modality in the primary care of CVDs (Maldonado-Garcia et al., 2022; Zhou et al., 2023). Additionally, a key benefit of the proposed approach is that it lends itself to explaining model predictions in both a global (across all instances) and local (instance-specific) sense, and thereby, provides insight into which retinal layers contain the most relevant information to identify risk of CVD.

Our results show that combining OCT-derived latent representations from both eyes with patient metadata yields the highest discriminative power for distinguishing between the CVD+ and CVD- groups (AUC: 0.75). While classifiers trained on left-eye data slightly outperformed those trained on right-eye data (AUC: 0.72 vs. 0.70 for LE-MTDT-RF and RE-MTDT-RF, respectively), the combined model achieved superior performance, suggesting that information from both eyes provides complementary features that improve predictive accuracy. Notably, left-eye features ranked highest in both global and local feature importance analyses. We hypothesize that this finding may be partly explained by systematic differences in image quality between eyes. In our cohort, left-eye scans exhibited higher average quality (see Supplementary Figure S5), likely because the UK Biobank protocol consistently acquired the left eye second, giving participants time to adapt to the procedure and potentially resulting in fewer motion artifacts. Although this acquisition order may introduce subtle biases favoring left-eye images, the improved performance of the combined model indicates that our findings are robust to these effects. Future studies using datasets with randomized or balanced acquisition sequences will help confirm that this performance difference is not primarily driven by protocol-related artifacts.

Additionally, in our age- and sex-matched cohort, the model correctly assigns a higher risk score to individuals who will experience a CVD event approximately 75% of the time, compared to only 60% for QRISK3. Importantly, this improved performance is achieved using a minimal set of demographic and clinical variables combined with retinal OCT-derived features, highlighting the potential of non-invasive retinal imaging to identify high-risk individuals more accurately while reducing false positives and unnecessary follow-up interventions.

Interestingly, our results suggest that choroidal morphology is a predictor of identifying patients at risk of CVDs, which is consistent with previous studies (Yeung et al., 2020) that have reported significant associations between choroidal characteristics and the risk of stroke and acute myocardial infarction. Given that the choroid has the highest flow per perfused volume of any human tissue and that there is growing evidence that changes in the choroid microvasculature can be indicative of systemic vascular pathology (Ferrara et al., 2021), our findings offer clinical interpretability to the predictions of our classifier. Currently, UK Biobank images are captured using a Spectral Domain (SD) OCT (Keane et al., 2016). SD-OCT images suffer significant light scattering at the choroid, which limits the resolution of this layer. Despite this limited resolution, it is encouraging to observe that the proposed approach focused on features within the choroidal layer to identify patients at risk of stroke or myocardial infarction. This suggests potential clinical applications, such as adapting standardized OCT acquisition protocols to enhance choroidal visibility (Chew et al., 2025). Techniques like enhanced depth imaging, swept-source OCT (which provides deeper penetration and better delineation of the choroid), or wide-field OCT angiography (OCTA; which offers complementary functional information by visualizing blood flow and quantifying vascular biomarkers) could be integrated into screening workflows. While swept-source OCT currently remains less widely available and more costly than spectral-domain OCT, ongoing technological advances and decreasing costs are likely to improve accessibility in both clinical and research settings. We hypothesize that combining multimodal retinal imaging (e.g. OCT, OCTA) with AI-based risk prediction may improve the classification performance of the proposed approach by capturing both anatomical and functional signatures of systemic vascular health, potentially improving the sensitivity and robustness of cardiovascular risk stratification (Copete et al., 2013; Runsewe et al., 2024; Germanese et al., 2024). In addition to the choroidal characteristics, our results indicated that the inner retinal layers contributed to the classification, including the Retinal Nerve Fiber Layer (RNFL), the Ganglion Cell Layer (GCL), and the Inner Plexiform Layer (IPL). These aspects of the neurosensory retina consist of retinal ganglion cells, their synapses with bipolar cells, and their axons. Regarding the choroid, thinning and defects in these layers have received extensive study in relation to established CVDs, but their role as predictors of future disease has received limited attention (Chen et al., 2023; Matuleviciūė et al., 2022). Mechanisms that may underpin the role of neurosensory retina morphology as a predictor of CVDs are yet to be elucidated, although it could be hypothesized that subclinical ocular circulatory pathology could explain morphological changes through local ischaemic damage (Chen et al., 2023), or neuronal degeneration could even occur through silent/subclinical cerebral ischaemic/vascular changes manifesting in the inner retina through transneuronal retrograde degeneration (Langner et al., 2022; Park et al., 2013).

Our study has several limitations. First, the analyses were conducted using data from the UK Biobank, a volunteer-based cohort known to have a “healthy volunteer” bias, with participants generally exhibiting lower smoking rates, healthier lifestyles, and lower prevalence of chronic conditions compared with the general population. This may have resulted in an underestimation of absolute CVD risk and may limit the generalizability of our results to more diverse or higher-risk populations (Fry et al., 2017). Additionally, althought we perfomed internal validation, we were unable to perform external validation, as no publicly available datasets currently combine 3D OCT volumes with longitudinal cardiovascular outcomes. Future research using clinical populations with broader demographic and health profiles, as well as independent external validation, is essential to confirm the robustness and generalizability of our findings. Such efforts could also explore the inclusion of additional clinical variables in predictive models, provided these variables are readily obtainable in typical eye care settings, thereby enhancing decision-making while maintaining practical applicability. Additionally, while our DL model demonstrates superior predictive performance compared to QRISK3, a direct comparison between the two is not entirely fair due to fundamental differences in their design and target population. QRISK3 has been extensively validated across large, diverse populations [e.g., QResearch (Hippisley-Cox et al., 2017), CPRD (Livingstone et al., 2021)] and is widely adopted in clinical practice. Our matched cohort may also reduce the predictive impact of key variables like age and sex (Ghosal and Sinha, 2022), further limiting direct comparability.

## 5 Conclusion

This study presents a predictive framework comprising a self-supervised representation learning approach based on a VAE and a Random Forest classifier, which effectively integrates multi-modal imaging (OCT imaging) and non-imaging (e.g. patient demographic and clinical variables) data, to identify patients at risk of stroke or myocardial infarction. Future work should explore multi-modal retinal imaging integration to enhance CVD prediction.

## Data Availability

Publicly available datasets were analyzed in this study. This data can be found here: https://www.ukbiobank.ac.uk/.
