# Supplementary material for: Predicting cardiovascular disease risk using retinal optical coherence tomography imaging
Source: Front Artif Intell. 2025 Nov 18;8:1624550. doi: 10.3389/frai.2025.1624550 (PMC12669110; doi:10.3389/frai.2025.1624550)
Supplement: Supplementary file 1 [file Data_Sheet_1.pdf]

S1 SUPPLEMENTARY MATERIALS

S1.1 Online Methods

The code used in this study is available on GitHub at the following address: Github/Predicting-CVD-using-OCT-images

In the Github account, in the folder named **Hyperparameter Results** there is an excel file called **grid_search_hyperparameters.xlsx** that contains the results from the grid search experiments conducted to determine the optimal parameters for our models (VAE, Random Forest, and Multilayer Perceptron). The file includes multiple sheets corresponding to different experiments. For example, the sheet `oct_vae_433` presents the grid search results for the VAE model with a data split ratio of 4:3:3 (training/validation/testing). Similarly, the sheet `oct_bemtdt_rf_433` provides the parameters and results from the grid search using the `sklearn.RandomizedSearchCV` library for the Random Forest model. Additionally, the file contains a comparison of the best results across various data split ratios, allowing for a clearer understanding of the performance differences. We also included some experiments we ran some experiments to select the optimal case:control ratio; sheets names are ratio_1_1, annd ratio_1_2; this was RF and BE-MTDT data.

S1.2 Figures

Figure S1 presents a bar plot illustrating the methods used to determine the onset date of stroke or myocardial infarction (MI) for the 612 CVD+ subjects involved in our classification task. The majority of cases, approximately 350, were attributed to hospital primary records, followed by hospital secondary records. The fewest cases were associated with death contributory records.

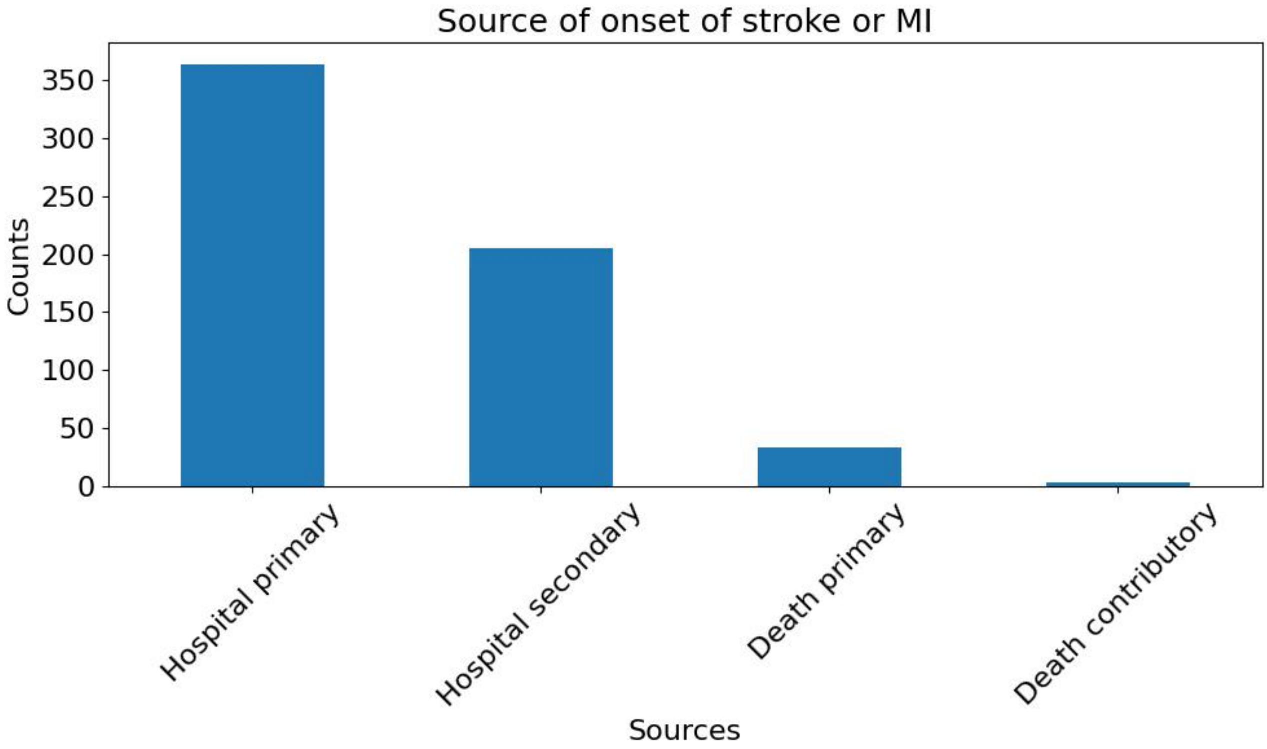

Figure S1. Bar plots showing the methods used to determine the onset date of stroke or MI for the 612 CVD+ subjects involved in our classification task (see details in (ukb, 2022))

Figure S2 and Figure S3 confusion matrices and precision–recall (PR) curves for all classifiers investigated. These analyses provide additional insight into classifier performance and class-specific prediction behavior.

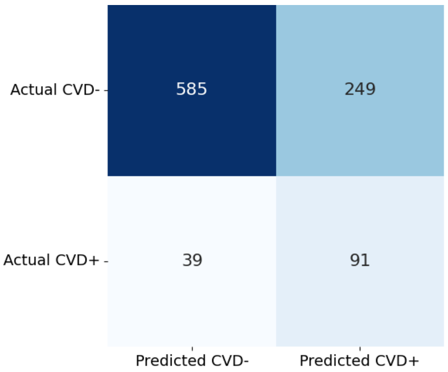

Figure S2a. BE-MTDT-RF

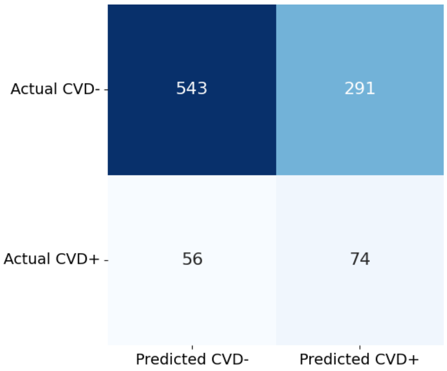

Figure S2b. BE-RF

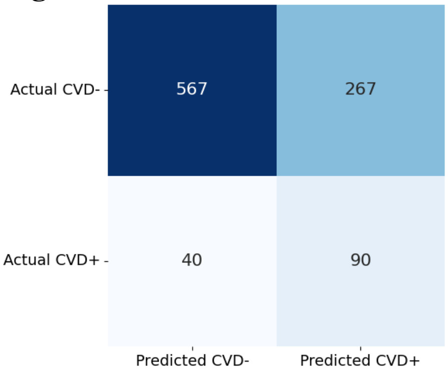

Figure S2c. LE-MTDT-RF

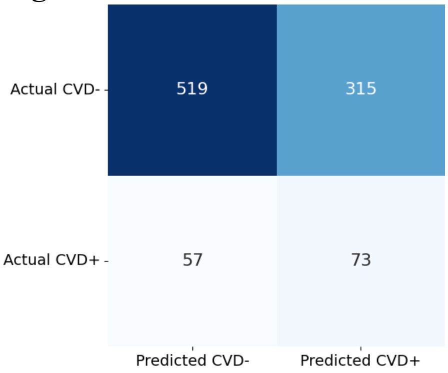

Figure S2d. LE-RF

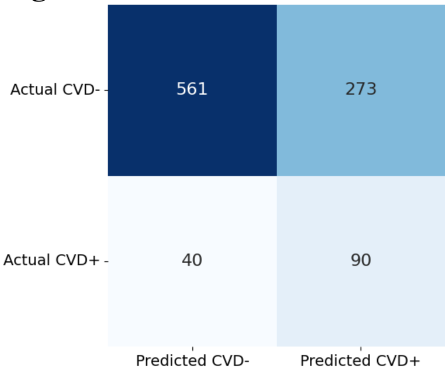

Figure S2e. RE-MTDT-RF

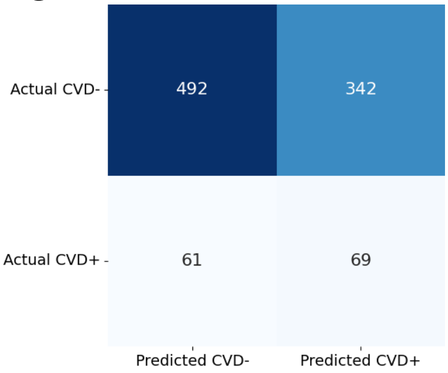

Figure S2f. RE-RF

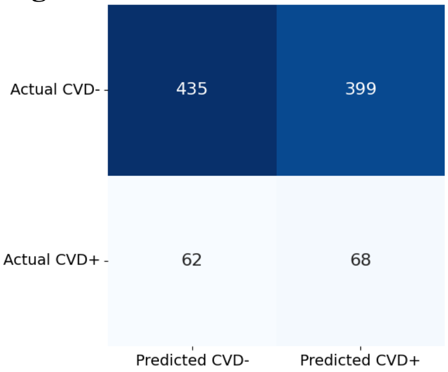

Figure S2g. MTDT-RF

Figure S2. Confusion matrices for the seven classifiers investigated. Predicted labels are shown on the x-axis and actual labels on the y-axis.

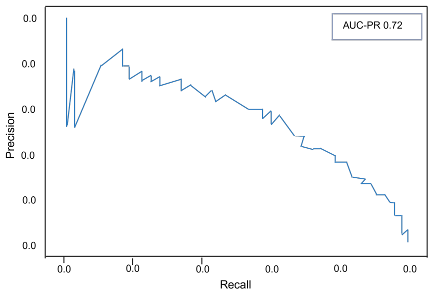

Figure S3a. BE-MTDT-RF

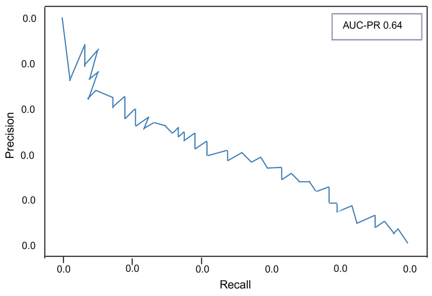

Figure S3b. BE-RF

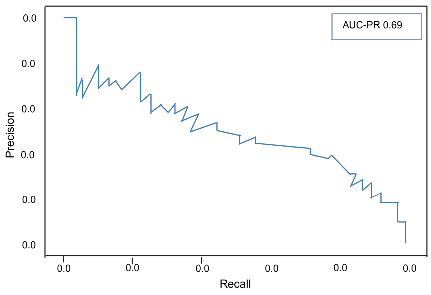

Figure S3c. LE-MTDT-RF

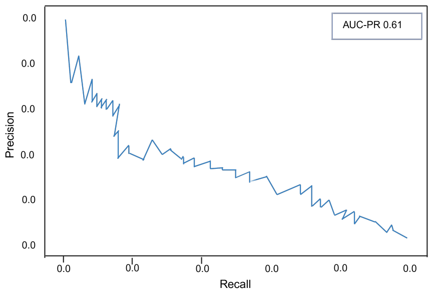

Figure S3d. LE-RF

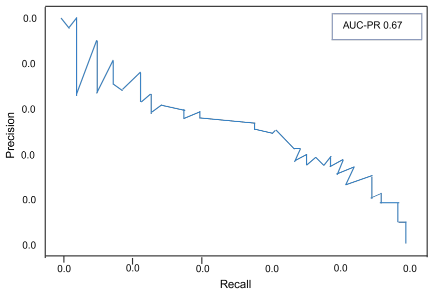

Figure S3e. RE-MTDT-RF

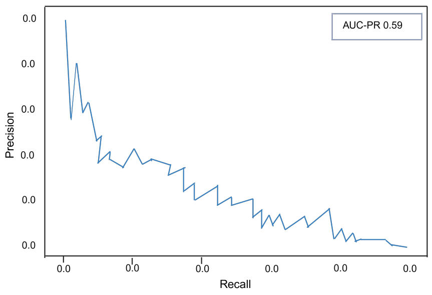

Figure S3f. RE-RF

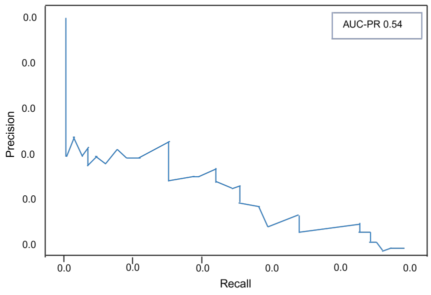

Figure S3g. MTDT-RF

Figure S3. AUC PR curves for the seven classifiers investigated. AUC-PR values were also AUC values are in each figure.

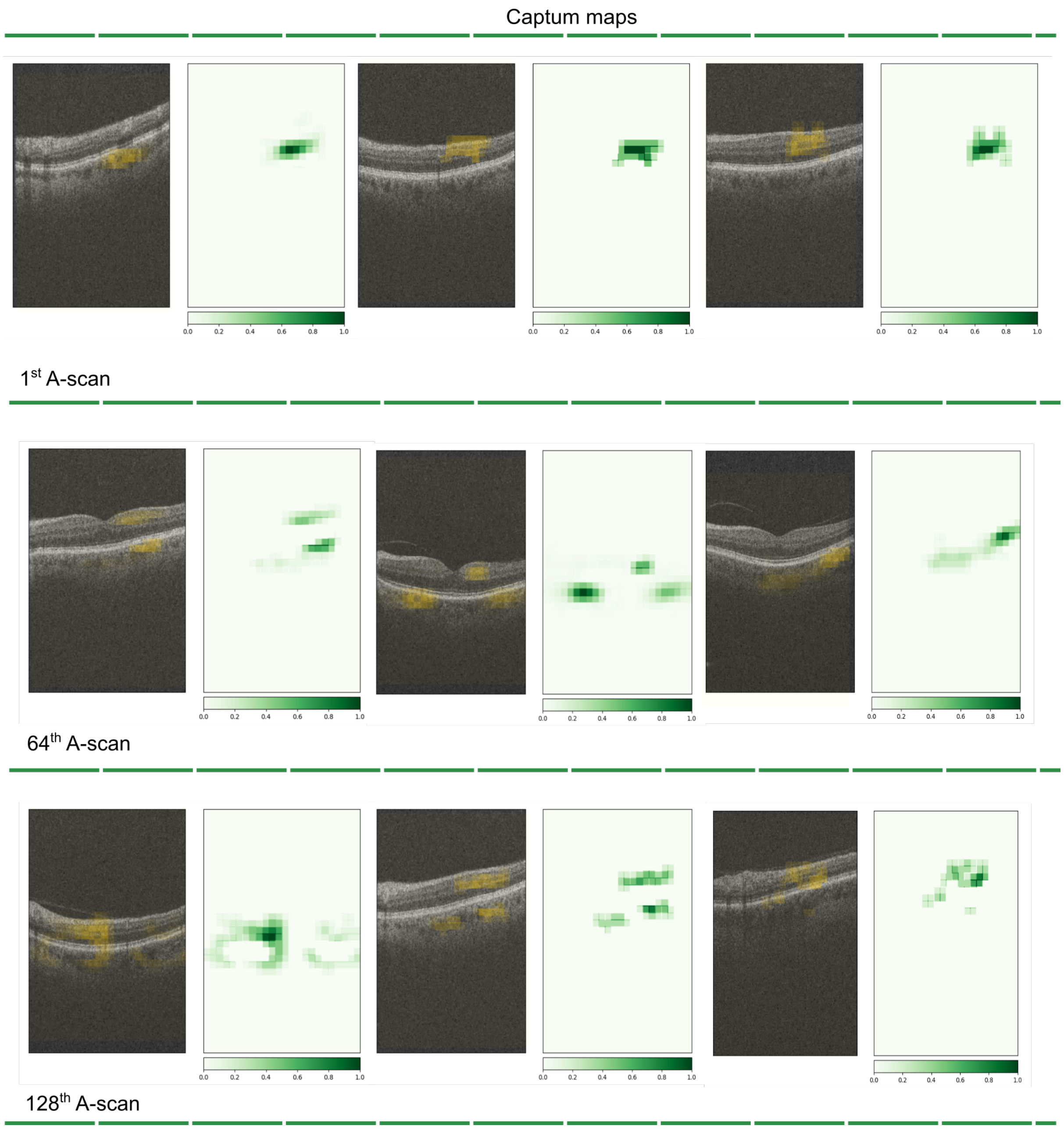

Figure S4. Captum images present three different B-scans for the left eye. The top row corresponds to the 1st B-scan, the middle image to the 64th B-scan, and the bottom row depicts the final B-scan. The yellow zones highlight regions of the oblation method when modifying the latent variable zl066.

The Quality Index is calculated as the product of two terms referred to as Intensity Ratio (IR) and Tissue Signal Ratio (TSR). The IR is akin to the signal-to-noise ratio (SNR), but rather than considering the maximum SNR value among all A-scans, it encompasses the entire image. Meanwhile, TSR represents the ratio of highly reflective pixels to those with lower reflectivity. Further details regarding the formula are provided in (Stein et al., 2006). The QI for both left and right eye OCT imaging is shown in Figure S5, with a quality threshold set at QI = 20.

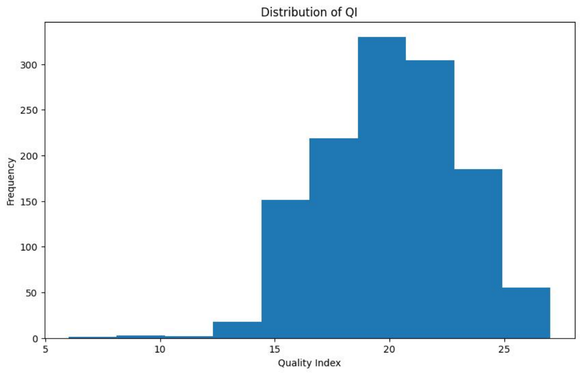

Figure S5a. Distribution of QI of the left eye

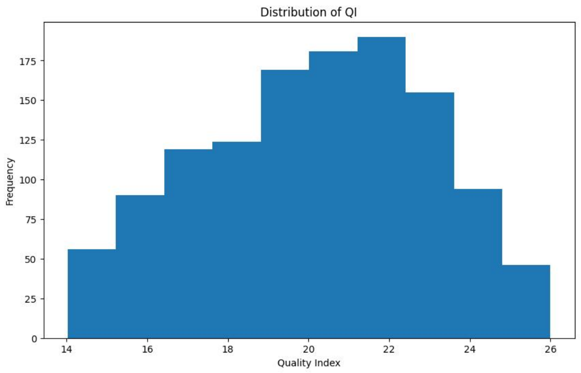

Figure S5b. Distribution of QI of the right eye

Figure S5. Comparation of the distribution of the Quality Index from both eyes.

S1.3 Tables

Hyperparameter	Tested Values	Optimal Value
CNN channels	[128, 256, 128]<br>[128, 256, 128, 64]<br>[128, 256, 128, 128]<br>[128, 256, 128, 128, 64]<br>[128, 256, 128, 128, 64, 64]<br>[128, 256, 128, 128, 64, 32]	[128, 256, 128, 128, 64, 64]
Batch size	4, 8, 16, 32	8
Learning rate	Log-scale: 10^{-1} to 10^{-6};<br>Multiples: 0.3, 0.5, 0.7, 0.9	0.0001
Weight decay	Log-scale: 10^{-1} to 10^{-6};<br>Multiples: 0.3, 0.5, 0.7, 0.9	0.0001
Latent space size	128, 256, 512, 1024	128
Train/Val/Test split ratio	4:3:3, 5:3:2, 5:2:3, 6:2:2	6:2:2

Table S1. Hyperparameter grid search for the VAE model. Tested values include architectural choices (CNN channels, latent space size), optimization parameters (learning rate, weight decay), and data splits. The optimal configuration was selected via 5 cross-validation.

Table S2 shows the architectural design of the Variational Autoencoder (VAE) used in our model, detailing the encoder and decoder components. It presents the structure of each network, including the layer types, configurations, and dimensional transformations that occur between layers. The encoder extracts features from the input RGB image, while the decoder reconstructs the image from the latent variables.

Encoder	Decoder
Input: $128 \times 224 \times 224$ (RGB image)	Latent variables, Fully Connected, Reshape
Conv2D(128, 3×3, stride=2×2, padding=1×1)<br>BatchNorm2d(128)<br>ReLU	Conv2D(64, 3×3, stride=2×2, padding=1×1)<br>BatchNorm2d(64)<br>ReLU
Conv2D(256, 3×3, stride=2×2, padding=1×1)<br>BatchNorm2d(256)<br>ReLU	Conv2D(64, 3×3, stride=2×2, padding=1×1)<br>BatchNorm2d(64)<br>ReLU
Conv2D(128, 3×3, stride=2×2, padding=1×1)<br>BatchNorm2d(128)<br>ReLU	Conv2D(128, 3×3, stride=2×2, padding=1×1)<br>BatchNorm2d(128)<br>ReLU
Conv2D(128, 3×3, stride=2×2, padding=1×1)<br>BatchNorm2d(128)<br>ReLU	Conv2D(128, 3×3, stride=2×2, padding=1×1)<br>BatchNorm2d(128)<br>ReLU
Conv2D(64, 3×3, stride=2×2, padding=1×1)<br>BatchNorm2d(64)<br>ReLU	Conv2D(256, 3×3, stride=2×2, padding=1×1)<br>BatchNorm2d(256)<br>ReLU
Conv2D(64, 3×3, stride=2×2, padding=1×1)<br>BatchNorm2d(64)<br>ReLU	Conv2D(128, 3×3, stride=2×2, padding=1×1)<br>BatchNorm2d(128)<br>ReLU
Latent variables, Fully Connected	Output: $128 \times 224 \times 224$ (Reconstructed RGB image)

Table S2. Architectural design of the Variational Autoencoder (VAE), showing the encoder and decoder structures with their respective layers, configurations, and dimensional transformations.

Hyperparameter	Tested Values	Optimal Value
Random state	0,1,2	BE-MTDT-RF: 1<br>BE-RF: 1<br>LE-MTDT-RF:2<br>LE-RF: 1<br>RE-MTDT-RF:0<br>RE-RF: 2<br>MTDT-RF: 1
Estimators	100–1000 (step 50)	BE-MTDT-RF: 300<br>BE-RF: 100<br>LE-MTDT-RF: 500<br>LE-RF: 500<br>RE-MTDT-RF: 700<br>RE-RF: 100<br>MTDT-RF: 400
Min samples split	10–500 (step 10)	BE-MTDT-RF: 210<br>BE-RF: 410<br>LE-MTDT-RF: 160<br>LE-RF: 360<br>RE-MTDT-RF: 260<br>RE-RF: 460<br>MTDT-RF: 360
Min samples leaf	10–500 (step 10)	BE-MTDT-RF: 60<br>BE-RF: 60<br>LE-MTDT-RF: 260<br>LE-RF: 10<br>RE-MTDT-RF: 10<br>RE-RF: 10<br>MTDT-RF: 260
Max features	0.25, 0.5, 0.75	BE-MTDT-RF: 0.25<br>BE-RF: 0.25<br>LE-MTDT-RF: 0.25<br>LE-RF: 0.25<br>RE-MTDT-RF: 0.25<br>RE-RF: 0.75<br>MTDT-RF: 0.5
Max depth	1, 2	BE-MTDT-RF: 1<br>BE-RF: 2<br>LE-MTDT-RF: 1<br>LE-RF: 1<br>RE-MTDT-RF: 1<br>RE-RF: 2<br>MTDT-RF: 1
Criterion	gini, entropy	BE-MTDT-RF: gini<br>BE-RF: gini<br>LE-MTDT-RF: entropy<br>LE-RF: entropy<br>RE-MTDT-RF: entropy<br>RE-RF: entropy<br>MTDT-RF: gini

Table S3. Hyperparameter grid search for RF models. Tested ranges and optimal values are shown for each variant (BE/LE/RE-MTDT-RF, BE/LE/RE-RF, and MTDT-RF). Step sizes are indicated in parentheses for ranged parameters.

Table S4 presents the list of variables used to calculate QRISK3, as obtained from (Li et al., 2019).

**Variables used in QRISK3**
Sex
Age
Atrial fibrillation
Atypical antipsy
Regular steroid tablets
Erectile disfunction
Migraine
Rheumatoid arthritis
Chronic kidney disease
Severe mental illness
Systemic lupus erythematosis
Blood pressure treatment
Diabetes1
Diabetes2
Weight
Height
Ethnicity
Heart attack relative
Cholesterol HDL ratio
Systolic blood pressure
Std systolic blood pressure
Smoke
Townsend

Table S4. List of Variables used to calculate QRISK3

Table S5 presents the chi-squared (χ^2) test results comparing BE-MTDT-RF with QRISK, LE-MTDT-RF, RE-MTDT-RF, MTDT-RF, BE-RF, LE-RF, and RE-RF, as well as BE-RF with LE-RF and RE-RF. The p-values indicate statistically significant differences between these classifiers (*$p < 0.005$). Table S6 presents the chi-squared (χ^2) test results comparing LE-MTDT-RF and RE-MTDT-RF (9.61×10^{-5}), and LE-RF and RE-RF (4.22×10^{-6}), with $p - values < 0.005$.

Classifier 1	Classifier 2	χ^2	P value
BE-MTDT-RF	QRISK	95.72	1.32×10^{-22}
BE-MTDT-RF	LE-MTDT-RF	15.21	9.261×10^{-05}
BE-MTDT-RF	RE-MTDT-RF	21.16	4.22×10^{-06}
BE-MTDT-RF	MTDT-RF	93.23	4.65×10^{-22}
BE-MTDT-RF	BE-RF	10.59	0.0011
BE-MTDT-RF	LE-RF	27.43	1.63×10^{-07}
BE-MTDT-RF	RE-RF	43.83	3.57×10^{-11}
BE-RF	LE-RF	21.16	4.22×10^{-06}
BE-RF	RE-RF	37.78	7.89×10^{-10}

Table S5. Comparison between various classifiers based on different configurations of eye data (both eyes, left eye, right eye) and metadata inclusion. The chi-squared (χ^2) values and associated p-values indicate the statistical significance of differences between these classifiers.

Classifier 1	Classifier 2	χ^2	P value
LE-MTDT-RF	RE-MTDT-RF	6	9.61×10^{-05}
LE-RF	RE-RF	17.06	4.22×10^{-06}

Table S6. Comparison of LE-MTDT-RF and RE-MTDT-RF, as well as LE-RF and RE-RF, using McNemar's Test (*$p < 0.005$).

Supplementary Table S7 provides the classification performance metrics evaluated on the same holdout set used for the experiments reported in Table 2. In this comparison, we assessed the performance of the BE-MTDT-RF model, which achieved superior results, against a Multilayer Perceptron (MLP) algorithm. For the MLP model, we conducted a comprehensive grid search to identify its optimal configuration, ensuring a fair comparison. Both models were evaluated using ocular data and metadata as input features. The BE-MTDT-RF model demonstrated superior performance across all evaluated metrics. We selected Random Forest (RF) as our classifier model due to its ability to mitigate overfitting (Breiman, 2001). Additionally, the feature importance analysis provided by RF offers valuable insights into the relative contributions of different features to the model's predictions, aligning with our exploratory objectives and potentially enhancing domain-specific understanding of the dataset. Moreover, RF is computationally less demanding than neural networks and does not require GPU resources for training, making it a more efficient choice for our analysis.

Classification metrics				
Modality	Accuracy (95% CI)	Sensitivity (95% CI)	Specificity (95% CI)	AUC (95% CI)
**BE-MTDT-RF**	**0.70 (0.67-0.73)**	**0.70 (0.67-0.73)**	**0.70 (0.67-0.72)**	**0.75 (0.72-0.78)**
MLP	0.65 (0.62-0.68)	0.64 (0.61-0.67)	0.65 (0.62-0.68)	0.72 (0.69-0.75)

Table S7. Comparison of classification metric results between our model employing both ocular data and metadata (BE-MTDT-RF) and the MLP algorithm. All metrics are reported as mean values with 95% confidence intervals (CI).
